# The Current Role of Neoadjuvant Chemotherapy in the Management of HER2-Positive, Triple-Negative, and Micropapillary Breast Cancer: A Narrative Review

**DOI:** 10.7759/cureus.49742

**Published:** 2023-11-30

**Authors:** Dhanashree Wankhade, Pankaj Gharde, Sushmita Dutta

**Affiliations:** 1 General Surgery, Jawaharlal Nehru Medical College, Datta Meghe Institute of Higher Education and Research, Wardha, IND

**Keywords:** neoadjuvant chemotherapy, breast cancer, breast conservation surgery, pathological complete response, axillary lymph nodes

## Abstract

Currently, the prevailing approach for managing breast carcinoma involves initiating neoadjuvant chemotherapy (NAC) as a part of the treatment regimen before surgery. NAC is being applied progressively in the therapeutic management of locally advanced breast carcinoma because of its capability to aid in surgery and facilitate the surgical treatment of patients who were once thought to be inoperable. Patients must be managed by a team of professionals from the start to the completion of the therapy. Pathological complete response (pCR), reduces the degree of recurrence of the disease and denotes the elimination of the tumor completely from the breast, it also indicates elimination of the tumor from the axillary lymph nodes. There is currently sufficient information to support the idea that patients would perform better if NAC resulted in a pCR. The administration of the same regimen of adjuvant therapy in neoadjuvant therapy provides women with similar improvements in overall survival. NAC offers potential benefits, such as enhancing the likelihood of breast conservation and broadening the scope of available surgical options. Based on how well they respond to neoadjuvant treatment, women receive a personalized prognosis evaluation. NAC has been proven to be very effective. However, patients can be resistant to medications easily which is not desirable for patients receiving this therapy going forward. In this review, we have discussed the purpose of managing patients with this therapy in locally advanced breast cancer. We have also discussed the various benefits of NAC as well as the application of different drugs, their advantages, and disadvantages that are given to the patient. The application of NAC in cases of human epidermal growth factor 2 (HER2) positive breast cancer and micropapillary breast cancer has also been discussed briefly in this review.

## Introduction and background

The recommended therapeutic approach for locally advanced breast cancer is neoadjuvant chemotherapy (NAC). It has been demonstrated that NAC helps in the conservation of the breast in individuals with locally advanced breast cancer who require mastectomy [1]. Additionally, individuals with operable breast tumors who received preoperative chemotherapy had a higher proportion of breast conservation surgery following NAC [2]. According to certain research looking at surgery for breast conservation following the treatment with NAC, locoregional rates of recurrence are below 10 percent [3]. Around 20 percent of breast cancer cases belong to the triple-negative subtype. They don't receive regular therapy [4].

Neoadjuvant therapy is a useful research tool that enables researchers to test new medicines as well as treatment regimens to evaluate the outcomes of chemotherapy on breast cancer. Evaluation of tumor response is necessary to acquire clinical information from clinical studies [5]. For the varied population of women, NAC is the established norm for care for locally advanced types of breast cancer. In order to anticipate medication response and support the creation of individualized treatment plans, serum biomarker levels are being studied more and more [6].

Any patient with a tumor larger than five centimeters, involving the skin, axillary lymph nodes that are immovable, or the lymph nodes that are involved unilaterally which includes supraclavicular as well as infraclavicular is considered to have a locally advanced breast tumor [7]. For individuals with breast cancer that is inflammatory in nature, NAC is the conventional treatment with the goal of achieving tumor resectability. NAC has benefits such as shrinking tumors, which makes inoperable tumors resectable, raising breast conservation surgery rates, allowing the medical care of micrometastases beforehand, and facilitating in vivo chemotherapeutic sensitivity testing [8].

NAC is being used to hasten the discovery of medications that are employed in the management of individuals suffering from triple-negative breast cancer (TNBC), it was developed for a varied subpopulation that is suffering from aggressive cancer and for whom no approved treatments are available [9]. Early assessment of drug effectiveness is made possible by measuring biomarkers based on tissue samples, gathering samples of the tissue affected by the tumor at the time of the operation, and employing radiological diagnostic techniques prior to NAC [9].

## Review

Search methodology

**Figure 1:**
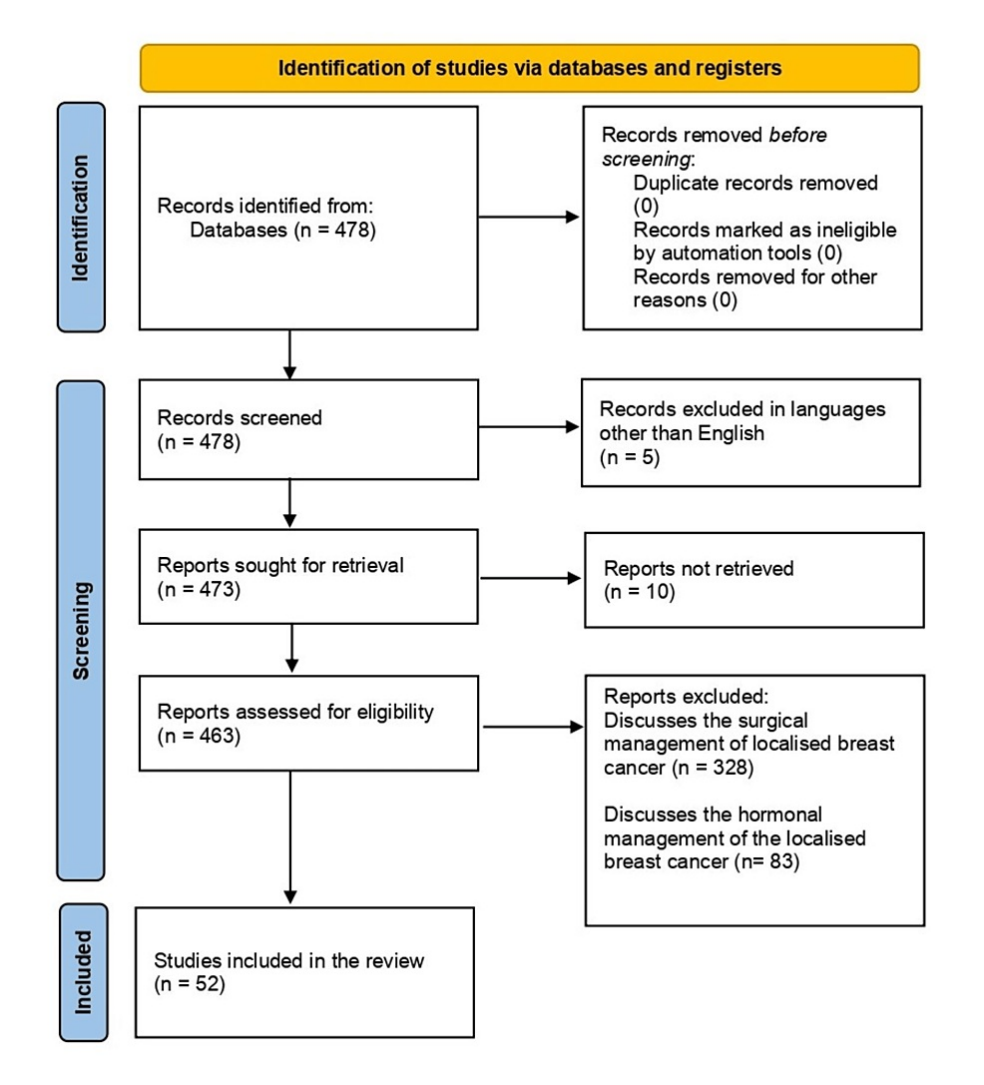
PRISMA flow diagram for screening and selecting articles for the use of neoadjuvant chemotherapy in locally advanced breast cancer PRISMA: Preferred Reporting Items for Systematic Reviews and Meta-Analyses

The eligibility criteria for this article included all review articles and case studies that discussed the application of NAC in locally advanced breast cancer. Review articles that discussed the application of NAC in TNBC as well as the advantages and disadvantages of using NAC. The literature search was conducted by all the authors. The PubMed electronic database was used for the literature search. A detailed search was done on PubMed and advanced Medical Subject Headings (MESH) terms such as neoadjuvant chemotherapy, locally advanced breast cancer, micropapillary breast cancer, and triple-negative breast cancer, were used interchangeably and in combination. The articles that were excluded were not retrievable and discussed either the application of chemotherapy after the surgical treatment or the application of NAC for cancers other than breast cancer. A total of 478 articles were found but only 52 were chosen to be included because it was determined that they were pertinent. These articles were selected following the Preferred Reporting Items for Systematic Reviews and Meta-Analyses (PRISMA) guidelines. The search process is demonstrated in Figure 1.

NAC for breast cancer refers to the therapeutic approach administered before surgical intervention. It was initially developed for those individuals who are suffering from locally advanced breast cancer, to downstage cancer that is not operable and minimizes the need for surgery, which also includes dissection of axillary lymph nodes [9].

NAC attempts to improve patient prognosis of breast cancers that are not operable into the ones that are operable, it also helps in breast conservation and helps in providing a pharmacological basis for the care of patients following the treatment [10]. Doxorubicin and epirubicin, both belonging to the anthracycline class are frequently employed with cyclophosphamide and fluorouracil as chemotherapeutic agents. NAC is now more efficient due to the emergence of taxanes and the substantial action of taxanes against advanced breast cancer [11]. The prognosis is good and hormone therapy works well for treating hormone receptor-positive breast carcinoma [12]. 

The significance of NAC in the treatment of breast tumors is growing steadily, and it is now considered necessary for high-risk cases [13]. Beyond attaining a pathological complete response (pCR), the advantages of the neoadjuvant approach include tumor downstaging, which enables surgical options that help in the conservation of breast, and evaluation of response, it offers useful prognostic data to enable the dosage of adjuvant medication to optimize the oncological prognosis [13].

NAC was primarily developed to manage individuals with inoperable breast cancer which is a locally advanced category of breast cancer. After adjuvant chemotherapy's advantages in breast carcinoma were established, it was also made available to patients with operable disease [14]. The ideal course of treatment is multidisciplinary and takes the tumor load and molecular type into consideration [14]. Surgery is frequently used as the main form of medical care because early-stage disease accounts for the bulk of presentations. Surgery may not always be the best starting point option for every patient, especially with early-stage disease [14]. Locally advanced breast cancer that was inoperable was the primary condition for which neoadjuvant therapy was employed [15]. The impact of NAC on individuals with operable carcinoma of the breast has subsequently been thoroughly studied. Preoperative NAC was considered a potential strategy to reduce the frequency of corrective surgery, broadening the concept that systemic therapy could enable certain surgically inoperable patients to undergo surgery [15].

Neoadjuvant treatment has been demonstrated to enhance the likelihood of breast conservation surgery in numerous studies of both chemotherapy and endocrine therapy, making it a feasible choice for patients with operable disease [15]. A multidisciplinary care team must supervise patients receiving neoadjuvant therapy. NAC may be utilized in patients with tumors that express the hormone receptor but lack the HER2 gene overexpression when a therapeutic choice can be made without access to surgical data [15].

The type of adjuvant treatment along with the drugs used in treatment is mentioned below in the following Table 1 [16].

**Table 1:** Adjuvant treatment given for breast carcinoma The above table demonstrates the adjuvant treatment given for breast cancer [16]. HER2: Human epidermal growth factor receptor 2, LH-RH: Luteinizing hormone-releasing hormone

Type of therapy	Biology	Drugs used in treatment
Hormone therapy	Estrogen receptor and progesterone receptor-positive premenopause	Tamoxifen with LH–RH agonist
		Aromatase inhibitor
Anti-HER2 therapy	HER2-positive	Chemotherapy along with Trastuzumab
		Chemotherapy along with Trastuzumab and Pertuzumab

Patients with axillary lymph node metastases face an elevated risk of recurrence. These patients should receive proper medications, which include anthracycline, docetaxel, and cyclophosphamide, or Adriamycin and cyclophosphamide after which docetaxel can be administered [16].

The new treatment that is being tested for its effectiveness through an ongoing trial of adjuvant treatment for breast cancer is mentioned below in the following Table 2 [16].

**Table 2:** The experimental treatment currently under evaluation in an ongoing adjuvant therapy trial for breast cancer The table given above demonstrates the experimental treatment currently under evaluation in an ongoing adjuvant therapy trial for breast cancer [16]. ER: Estrogen receptor, HER2: Human epidermal growth factor receptor 2

Subtype	Drug
ER-positive	Pembrolizumab, Abemaciclib, Palbociclib, Olaparib
ER-negative HER2-positive	T-DM1 (Transtuzumab emtansine)
Triple-negative	Atezolizumab, Olaparib

Neoadjuvant systemic chemotherapy

NAC was primarily employed as the therapeutic approach for locally advanced breast cancer without metastatic spread. The recent data from clinical trials have significantly changed the goals of NAC [17]. The goal of NAC is not just to elevate the number of breast-conserving surgeries, but also to improve precision medicine [17]. According to cancer biology, the selection of the regimen seeks to get the maximum anti-cancer effect. Patients with breast cancer who are HER2-positive take anti-HER2 medications [18].

Neoadjuvant chemotherapy

Patients who receive NAC have a higher probability of breast conservation than those who receive adjuvant treatment [19]. When it comes to lowering the likelihood of distant relapse and mortality, NAC is just as effective as adjuvant chemotherapy. However, when compared with adjuvant chemotherapy, NAC is linked to an increased chance of local relapse, which may be at least partially explained by the application of breast conservation surgery following NAC [19]. It is crucial to think about methods to reduce the risk of local relapse following breast conservation tumors reduced by NAC, such as accurate tumor localization, thorough pathological evaluation, and suitable irradiation [19]. The typical NAC regimen consists of an Adriamycin and cyclophosphamide (AC) and a taxane. The pCR rate in HER2-positive individuals is raised by anti-HER2 medications [20]. Notably, the concurrent use of trastuzumab and pertuzumab has demonstrated enhanced effectiveness while minimizing the occurrence of severe side effects [21].

NAC has been proven to be efficient, However, the development of drug resistance can pose a significant challenge for patients undergoing this therapy, which is not an ideal outcome [22]. Resistance to medications is a major factor in the failure of NAC, and it is also one of the most difficult issues that doctors are now dealing with [22]. Due to the ability to individualize NAC based on the pCR status following surgery, neoadjuvant therapy has become an essential factor in providing appropriate care to women with stage 2 or stage 3 tumors [23]. Newly approved medications for HER2-positive breast tumors like tucatinib, and trastuzumab-deruxtecan, as well as immunotherapy combinations, are being studied [23]. NAC trials proposed that survival would be improved by early treatment of subclinical micrometastases. NAC was once only used for locally advanced cancers; now, it is often used for operable breast tumors, and in these clinical trials [24]. For those who treat breast cancer, the effects of a pCR are quite important. Adjuvant therapy depends on the consequences of a precise assessment of the remaining disease in the breast as well as the axilla [24]. Many of the studies that have suggested that obtaining a pCR through NAC in breast cancer in recent years have relied primarily on magnetic resonance imaging [25].

The tumor that is still present following NAC vary in their pathologic appearance. In some cases, the histologic appearance of the initial tumor before and after treatment is the same. Most frequently, the tumor becomes less cellular overall and breaks into individual cells. The cells are larger in size and have several nuclei, a huge amount of cytoplasm, and pleomorphism [26]. In some categories of breast cancer patients, NAC use is increasing and is quickly taking over as the preferred method of therapy. The most reliable method for assessing the tumor effect of NAC in surgical tissues is still detailed histopathological assessment [26]. A direct line of contact with the medical pathologist is necessary for the examination of samples following NAC since it presents additional challenges and differs from the assessment of samples from patients who were not treated in this way. Therefore, a precise diagnostic evaluation is essential for informing clinical judgments, follow-up care, and prognosis [26]. Locally progressed cases were thought to be a sign of an operable tumor in the early days of NAC due to its capacity to reduce the staging of tumors [27]. NAC was initially pursued in order to improve drug development. The importance of NAC has led to it being the preferred method for the majority of triple-negative breast tumors [28].

Application of neoadjuvant chemotherapy in triple-negative breast cancer

pCR is the short-term objective of neoadjuvant treatment since it is linked to prolonged survival outcomes in individuals suffering from TNBC. Adding pembrolizumab to NAC increased its benefits [29]. NAC plus pembrolizumab, the use of pembrolizumab after surgery, efficiently increased overall survival compared to the management of the patients in whom NAC is given alone who are suffering from TNBC [29]. NAC along with adjuvant therapy's long-term objective is to stop the spread of metastasis [29]. Currently, it appears that the immune checkpoint inhibitor treatment may be more beneficial for the subpopulation that is positive for programmed cell death ligand 1 [30]. Pembrolizumab recently showed a better response for TNBC in the early stages. This positive outcome raises the possibility of the drug's approval for utilization in neoadjuvant settings [30].

NAC is applied in the management of numerous individuals with TNBC. With NAC, about one-third of patients will have positive results and a pCR. The chance of relapse is considerable for two-thirds of individuals, who will still have disease [31]. The NAC drug platinum is crucial for the treatment. TNBC patients who received carboplatin and nab-paclitaxel experienced a pathological complete response that was well tolerated and very effective [32]. The predictive value of pCR in HER2-positive and TNBC was later verified by neoadjuvant trials aimed at targeted therapies and NAC and is presently the optimal level of care for stage 2 and stage 3 HER2-positive cases of breast tumors [33]. Patients who have pCR after the treatment have a markedly improved likelihood of survival, whereas individuals who still have a disease that is invasive have a greater chance of recurrence following treatment [34]. Even though there is a strong association between attaining a pCR and enhanced survival rates, approximately 30 percent of patients with TNBC achieve pCR after receiving cyclophosphamide [35].

Bevacizumab has been demonstrated to elevate the number of TNBC individuals with pCR [36]. Due to the absence of survival data in the majority of research, the relationship between NAC and longevity results was not examined. However, some randomized control trials are still in progress, and a secondary evaluation emphasizing lasting survival advantages may be published soon. The impact of these treatments on medical outcomes will thus be included through an additional upgraded systematic review [37]. Adjuvant chemotherapy is the exclusive systemic therapeutic option for TNBC patients because anti-HER 2 receptor as well as endocrine therapy are not appropriate for them [38].

Only around one-third of patients on conventional anthracycline- and taxane-based regimens had pCR at surgery, despite having markedly improved outcomes than individuals with residual invasive disease after NAC [39]. The pCR rate was increased preoperatively by the combination of Taxane and Adriamycin-cyclophosphamide [40]. Veliparib has shown marked progress in survival, especially in individuals suffering from TNBC, and in individuals with metastatic breast tumor [41]. There was marked progress in the response in patients when carboplatin and veliparib were given with paclitaxel rather than paclitaxel alone [42]. pCR is improved by adding carboplatin to anthracycline chemotherapy, and carboplatin given along with taxane regimens also produces better pCR rates in patients having TNBC [43]. Recently, in the method of management of locally advanced breast tumors, therapeutic agents such as docetaxel, pyrotinib, and trastuzumab proved to be very effective and increased the rate of survival outcomes [44].

It has been demonstrated that pCR predicts long-term therapeutic benefits following NAC [45]. In a real-life scenario, thorough histopathological analysis of the tumor response to attaining pCR following NAC was linked to practically significant gains in survival outcomes. The overall results show that pCR is an acceptable substitute for the primary objective in both clinical research and situations based on population [45]. The number of patients increases dramatically when neoadjuvant carboplatin is administered with a regimen of taxane [46].

Application of neoadjuvant chemotherapy in HER2-positive breast cancer

NAC proved beneficial for individuals who are suffering from HER2-positive breast carcinoma. The therapeutic approach to the individual suffering from HER2-positive breast cancer at an early stage involves treatment with therapeutic agents that include trastuzumab and neratinib [47]. When trastuzumab was included in the NAC regimen, it markedly increased the percentage of individuals with HER2-positive breast tumors, essentially doubling the success rate for the patients who attained a pCR [48]. Incorporating pertuzumab with trastuzumab in neoadjuvant treatment is well-tolerated, leading to a notable increase in the rate of achieving a pCR when compared to using trastuzumab [48]. 

Tyrosine kinase inhibitors like pyrotinib also proved to be efficient in individuals suffering from breast cancer which is HER2-positive [49]. In the neoadjuvant trastuzumab and lapatinib treatment optimization (NeoALTTO) trial, it appears that women suffering from HER2-positive breast carcinoma experienced a significant improvement in achieving a pCR when treated by adding lapatinib to trastuzumab as compared to using only one anti-HER2 agent for the treatment [49].

Application of neoadjuvant chemotherapy in micropapillary breast cancer

Invasive micropapillary carcinoma (IMPC) is a variant of breast tumors known for its potential aggressiveness and an early tendency to involve the lymphatic system [50]. The indications for neoadjuvant treatment in IMPC appear to remain largely unchanged. IMPC tumors often exhibit overexpression of mucin-4, a glycoprotein that can effectively mask the target epitope of trastuzumab. Consequently, this molecular interaction can lead to treatment resistance and reduced overall survival rates among IMPC patients [51].

Currently, the presence of micropapillary features within invasive ductal carcinoma (IDC) does not influence the prognosis or guide decisions regarding adjuvant treatment strategies. Comprehensive clinical observations are necessary to uncover the underlying mechanism and formulate personalized treatment approaches [52]. A summary of all the articles included in the review is listed in Table 3.

**Table 3:** The above table demonstrates the summary of the articles included in the review TNBC: Triple-negative breast cancer, HER2: Human epidermal growth factor receptor 2, NAC: Neoadjuvant chemotherapy, AC: Adriamycin and cyclophosphamide, BRCA: Breast cancer gene, DNA: Deoxyribonucleic acid, IMPC: Invasive micropapillary carcinoma, MUC4: Mucin-4, pCR: Pathological complete response

Authors	Year	Findings
Zhou and Li [1]	2016	Breast conservation can be a secure option for individuals with breast tumors who respond positively to NAC.
Wolmark et al. [2]	2001	Individuals who had operable breast tumors and underwent preoperative chemotherapy showed a higher likelihood of undergoing breast conservation surgery after receiving NAC.
Chen et al. [3]	2004	After breast conservation surgery following NAC treatment, the rates of locoregional recurrence remain under 10 percent.
Hurley et al. [4]	2013	Approximately one-fifth proportion of breast carcinoma cases are classified as triple negative, and they do not typically receive standard therapy.
Romero et al. [5]	2013	Assessing tumor response is essential for obtaining clinical insights from clinical studies.
Nolen et al. [6]	2008	The increasing focus on studying serum biomarker levels is aimed at predicting medication responses and facilitating the development of personalized treatment plans.
Luangdilok et al. [7]	2014	A patient with a tumor exceeding five centimeters, skin involvement, and immobile axillary lymph nodes is classified as having locally advanced breast cancer.
Sachelarie et al. [8]	2006	The conventional strategy for achieving the resectability of tumors is through NAC.
Tufano et al. [9]	2021	NAC is being employed to expedite the development of treatments for individuals afflicted with TNBC.
An et al. [10]	2021	NAC contributes to breast preservation and offers a pharmacological foundation for post-treatment patient care.
Shen et al. [11]	2021	The efficacy of NAC has increased due to the introduction of taxanes and their significant impact on advanced breast cancer.
Iwamoto et al. [12]	2020	TNBC typically presents a challenging overall prognosis; however, it demonstrates favorable responses to HER2-targeted therapy and chemotherapy.
Provenzano [13]	2021	The benefits of the neoadjuvant approach encompass tumor downstaging allowing for surgical choices that promote both axillary and breast preservation.
Tse et al. [14]	2021	The advantages of the neoadjuvant approach include tumor size reduction, and facilitating surgical options that support preservation of both the axillary and breast.
Korde et al. [15]	2021	The use of preoperative NAC was seen as a prospective approach to decrease the need for additional corrective surgeries.
Shien and Iwata [16]	2020	Patients at high risk should be administered appropriate medications, such as anthracyclines, Taxotere, and cyclophosphamide.
Slamon et al. [17]	2011	The objective of NAC extends beyond enhancing the frequency of breast conservation surgeries.
Buzdar et al. [18]	2005	Individuals diagnosed with HER2-positive breast tumors receive anti-HER2 medications.
Asselain et al. [19]	2018	Patients undergoing NAC have a greater likelihood of preserving their breasts compared to those receiving adjuvant treatment.
von Minckwitz et al. [20]	2012	The typical NAC regimen consists of an AC and a taxane.
Gianni et al. [21]	2012	Pertuzumab and trastuzumab together have increased effectiveness without having serious side effects.
Zhao et al. [22]	2020	Medication resistance represents a significant factor contributing to the challenges in the effectiveness of NAC, and it poses one of the most formidable issues currently confronting medical professionals.
Harbeck [23]	2022	The use of neoadjuvant therapy has evolved into a crucial element in delivering the right care for women with stage 2 or stage 3 tumors.
Fisher [24]	2022	The choice of adjuvant treatment hinges on the result of a meticulous evaluation of any residual disease in the breast.
Pesapane et al. [25]	2022	In recent years, prediction models for achieving a pCR with NAC in breast cancer have predominantly utilized magnetic resonance imaging.
See and Siziopikou [26]	2022	Following NAC, the tumor becomes less cellular overall and breaks into individual cells.
Oikawa [27]	2020	Locally progressed cases who received NAC were thought to be a sign of an operable tumor.
Leon-Ferre et al. [28]	2021	The significance of NAC has made it the favored approach for the majority of individuals suffering from TNBC.
Schmid et al. [29]	2022	The inclusion of pembrolizumab alongside NAC resulted in an augmentation of its advantages.
Kwapisz [30]	2021	Recently, pembrolizumab exhibited a more favorable response in early-stage TNBC.
Radovich et al. [31]	2020	NAC gives positive results and a pCR in about one-third of patients suffering from TNBC.
Yuan et al. [32]	2021	TNBC patients who were treated with carboplatin and nab-paclitaxel achieved a well-tolerated and highly effective pCR.
Torrisi et al. [33]	2021	Subsequent neoadjuvant trials focused on targeted therapies confirmed the predictive significance of pCR in HER2 positive cases.
Cortazar et al. [34]	2014	Individuals who attain a pCR following this treatment have substantially enhanced prospects for survival.
Liedtke et al. [35]	2008	Approximately 30 percent of patients having TNBC experience pCR following cyclophosphamide.
Earl et al. [36]	2015	Studies have demonstrated that bevacizumab elevates the rate of TNBC patients achieving a pCR.
Li et al. [37]	2019	TNBC is more frequently associated with BRCA mutations.
Joensuu and Gligorov [38]	2012	Adjuvant treatment is the exclusive systemic therapeutic option for TNBC patients.
Biswas et al. [39]	2017	One-third of patients on conventional anthracycline and docetaxel had pCR at surgery.
Rastogi et al. [40]	2008	The preoperative combination of taxane and AC significantly elevated the rate of achieving a pCR.
Rodler et al. [41]	2016	Veliparib has shown significant improvements in survival rates in TNBC patients.
Geyer et al. [42]	2022	Administrating carboplatin and veliparib alongside paclitaxel led to a notable enhancement in patient responses.
Sharma et al. [43]	2021	Carboplatin given along with taxane regimens also produces better pCR rates in patients having TNBC.
Wu et al. [44]	2022	Neoadjuvant docetaxel, pyrotinib, and trastuzumab considerably increased the pCR rate.
LeVasseur et al. [45]	2020	A comprehensive histopathological examination of the tumor response leading to the attainment of a pCR after NAC was correlated with substantial improvements in survival outcomes.
Minckwitz et al. [46]	2014	The patient count experiences a notable surge when neoadjuvant carboplatin is combined with a taxane-based regimen.
Sharma et al. [47]	2020	The therapeutic approach for HER2-positive breast tumors involves therapeutic options that include trastuzumab and neratinib.
Takada and Toi [48]	2020	Incorporating pertuzumab with trastuzumab in neoadjuvant chemotherapy is well-tolerated by patients.
Yin et al. [49]	2022	Pyrotinib proved beneficial in individuals who are HER2-positive.
Zhang et al. [50]	2020	IMPC is potentially aggressive and may exhibit early involvement of the lymphatic system.
Verras et al. [51]	2022	IMPC tumors often exhibit overexpression of MUC4, a glycoprotein that can effectively mask the target epitope of trastuzumab.
Guan et al. [52]	2020	Comprehensive clinical observations are necessary to formulate personalized treatment approaches.

## Conclusions

NAC has been demonstrated to elicit a favorable tumor response in individuals suffering from locally advanced breast cancer. This enhanced response to therapy has not only broadened the spectrum of surgical options but also yielded improved survival rates for patients. Significantly, a growing population of individuals diagnosed with breast carcinoma are opting for breast conservation surgery after receiving NAC. Moreover, those who achieve a pCR after NAC experience more favorable recovery outcomes. The likelihood of pCR is enhanced when carboplatin is administered in combination with anthracycline chemotherapy, and combining carboplatin with taxane shows promising results, particularly in TNBC patients. Therapeutic options for HER2-positive individuals that involved the addition of pertuzumab to trastuzumab in the NAC regimen are well-tolerated, resulting in significant improvement in the rate of achieving a pCR. Pyrotinib also proved to be effective in HER2-positive cases. To better understand the underlying mechanism and develop treatment options for individuals with IMPC, extensive clinical observation is needed. More research is needed to comprehend the impact of NAC in treating individuals with IMPC.
